# Associations between age at first calving, productivity, survival, and economic performance in US Holsteins

**DOI:** 10.3168/jdsc.2026-1103

**Published:** 2026-08-24

**Authors:** Michael W. Overton

**Affiliations:** Zoetis Inc., Kalamazoo, MI 49007

## Abstract

Age at first calving (AFC) influences raising costs, future milk production, cow survivability, and herd profitability. This retrospective observational study examined associations between AFC and productivity, survival, and economic performance through 305 d of the second lactation in US Holstein dairy cows. Data from 74 US dairies included 128,252 Holstein cows born from January 2020 to June 2021 with AFC of 21 to 27 mo. Production outcomes included projected 305-d milk in first lactation (P305M), cumulative milk through 305 d of first lactation (CUML1), and cumulative milk through 305 d of second lactation (CUML1&2). Replacement risk, stillbirth risk, and cumulative DIM were also assessed. Linear mixed models (with herd and mo-yr of birth as random effects) and multivariable logistic regression were used. Although P305M was highest at 26 mo AFC, both CUML1 and CUML1&2 were maximized at 23 mo, driven partly by lower removal risks in younger-calving cohorts. First-lactation replacement risk increased progressively from 17% (21 mo AFC) to 35% (27 mo AFC). An integrated economic model incorporating milk revenue, calf value, feed, replacement, and raising costs showed comparable net income among 21- to 23-mo AFC cohorts, with a progressive decline beginning at 24 mo. Despite potential first-lactation production advantages for older-calving heifers, delaying first calving beyond 23 mo carries meaningful economic consequences. Results support targeting 22 to 23 mo AFC in US Holstein herds, provided adequate growth and BW at first calving are achieved.

## Graphical Abstract

**Figure:**
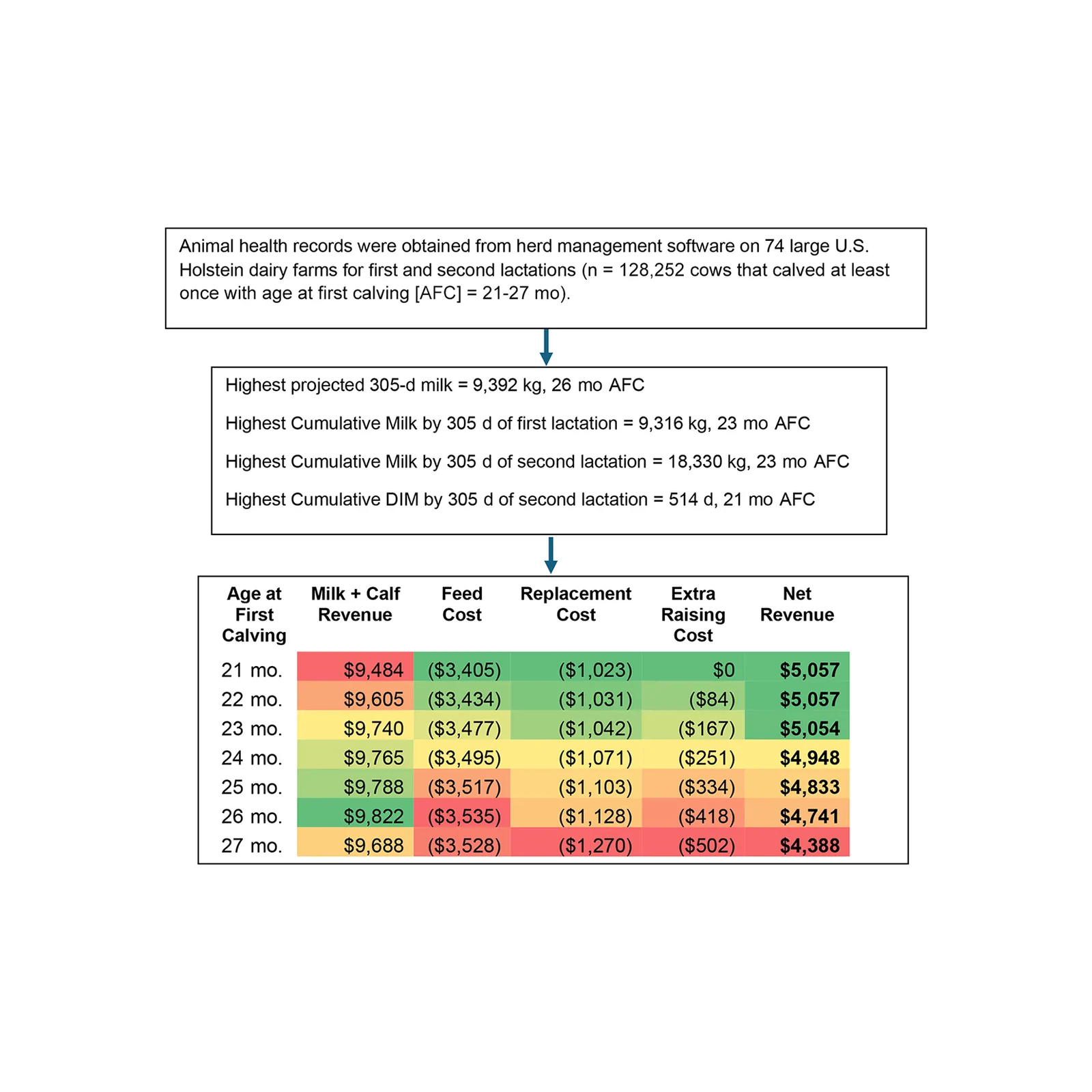

Age at first calving (**AFC**) is linked to replacement economics, first-lactation milk yield, longevity, and long-term herd productivity (Tozer and Heinrichs, 2001; Ettema and Santos, 2004; Atashi et al., 2021). Replacement costs are typically the second or third largest expense after feed and AFC is a key driver (Tozer and Heinrichs, 2001). Average US heifer-raising costs range from $2,200 to >$2,600, with marginal costs of $1,800 to $2,100 per animal (Overton and Eicker, 2022a). Earlier AFC can reduce the number of heifers needed in the replacement pipeline, and Tozer and Heinrichs (2001) estimated that each 1-mo reduction in AFC decreased total replacement program cost by 4.3%. However, AFC is also associated with dystocia, stillbirth risk, milk yield, fertility, and survival, and reported relationships are often inconsistent (Ettema and Santos, 2004; Atashi et al., 2021; Marcondes et al., 2025). Thus, optimal AFC may vary depending on the outcome measured and length of follow-up (i.e., first lactation or over multiple potential lactations).

Age at first calving reflects growth, health, breeding readiness, breeding management, heifer fertility, genetics, and management preferences. Globally, AFC has ranged from approximately 24 to >29 mo but is trending toward current Holstein recommendations of 23 to 24 mo (Ettema and Santos, 2004; Sawa et al., 2019; Van Eetvelde et al., 2020). Producers influence AFC mainly through nutrition and reproductive management, especially age at first insemination. Higher pre- and postweaning growth rates support earlier AFC and greater milk production when reproduction is managed accordingly (Soberon et al., 2012; Hurst et al., 2022). First insemination timing may be based on age, weight, height, or combinations. With daily gains of ∼0.81 to 0.88 kg/d and a breeding target of 55% of 57% of expected mature BW, Holsteins are typically first inseminated at 13 to 15 mo (Fox et al., 1999; Duplessis et al., 2015).

Target size at first calving is typically 82% to 85% of mature body weight (**MBW**) at 1 to 3 DIM (Kertz et al., 1997; Han et al., 2021; Lauber and Fricke, 2023). Larger size at first calving is associated with higher first-lactation productivity (Van Amburgh et al., 1998; Handcock et al., 2019). The second-lactation target is ∼92% MBW; lighter heifers are more likely to partition nutrients toward growth rather than production (Fox et al., 1999; Olson et al., 2010). Heifers growing faster before calving (∼1.0 vs. 0.85 kg/d) while still calving at 23 to 24 mo may enter second lactation closer to mature size, reducing nutrient partitioning toward growth.

Breeding eligibility windows also influence AFC. Historically, breeding windows extended to 6 inseminations or 6 mo (or longer; Van Amburgh et al., 1998; Mourits et al., 2000). A recent University of Idaho survey (n = 18 farms) found that 39% of respondents limited breeding to 90 to 120 d, whereas the remainder allowed 150 d or more (Joe Dalton, University of Idaho, Caldwell, ID, personal communication, May 2026). With a voluntary waiting period of 13 to 15 mo, allowing ≥6 mo of breeding opportunity will result in some heifers calving at ≥28 mo, which is generally suboptimal.

Evidence suggests older AFC can increase first-lactation yield, but often with trade-offs. Atashi et al. (2021) reported higher partial and completed 305-d yields with increasing AFC, alongside longer first-calving intervals. Marcondes et al. (2025) found more dystocia with AFC >26 mo, but extremes on either end may elevate risk. Conversely, earlier AFC achieved through adequate growth and good fertility can improve lifetime productivity (Cooke et al., 2013). Ultimately, optimal AFC is a lifetime-efficiency question, and conclusions depend on the outcome and timeframe evaluated.

Survivability is central to determining the economically optimal AFC. Mourits et al. (2000) identified an optimum averaging 20 to 21 mo at a specified BW, whereas Ettema and Santos (2004) concluded that managing for 23 to 24.5 mo maximized adjusted first-lactation income. Reducing AFC can accelerate positive cash flow, reduce on-farm heifer inventory, and improve discounted net returns when biological constraints are respected. The hypothesis of this project was that optimal AFC would vary depending upon production measure and follow-up period evaluated.

The objective of this retrospective observational study was to examine associations between AFC and productivity and survival through the end of second lactation, and to estimate economic costs and opportunities. Herd records were extracted from DairyComp (Valley Agricultural Software, Madison, WI) from a convenience sample of 74 US dairies that granted permission for anonymous use of their records. Eligible animals were Holsteins born January 1, 2020, to June 30, 2021, that first calved February 1, 2021, to December 21, 2023, with an AFC of 21 to 27 mo. Cows sold for dairy purposes or as genomic culls were excluded (n = 7,720). First- and second-lactation records (when present) were joined into a single dataset.

Milk production was evaluated 3 ways: projected 305-d milk in the first lactation (**P305M**), cumulative milk by 305 d of the first lactation (**CUML1**), and cumulative milk by 305 d of the second lactation (**CUML1&2**). Projected 305-d milk estimates projected production over a standardized 305-d lactation (∼10 mo) by combining linear interpolation between test-day records with extension factors from published tables (e.g., USDA Council on Dairy Cattle Breeding). These factors are based on Wood function lactation curves adjusted for DIM at last test, parity, breed, and age at calving. The CUML1 and CUML1&2 values were estimated using a custom DairyComp item that estimates total milk (using a similar approach to P305M) from calving to herd removal or the endpoint, whichever occurred first (e.g., cows leaving at 250 DIM contribute 0 milk from 251 to 305 d). For CUML1&2, cumulative milk through 335 d of first lactation was added to cumulative milk through 305 d of second lactation. The 335-d endpoint approximates average time to dry-off and avoids upward bias from a smaller subset of terminal, nonpregnant lactations. Cumulative days in milk (**CUMDIM**) summed lactating days of 335 d for first and 305 d for second lactation, consistent with CUML1&2.

Candidate explanatory variables included routinely recorded, biologically relevant factors and 2-way interactions. Pneumonia incidence as a heifer (**PNEU**: 0, 1, 2, or 3+ cases), stillbirth (yes or no), and twins at first calving (yes or no) were offered to all models; mastitis was not included due to longer time-at-risk bias in surviving cows. Age at first calving (mo) was modeled as a nominal variable rounded to the nearest whole mo (e.g., 22 mo = 21.5–22.4). Dam parity during gestation (**DLACT**) had 5 levels (0, 1, 2, 3, and 4+). Herd and mo-yr of birth were included. Inconsistently available variables such as birth weight, dystocia score, diarrhea incidence, weight at first calving, and genomic values were excluded.

Analyses used JMP Pro 19.0.4 (JMP Statistical Discovery LLC, Cary, NC). Linear mixed models with herd and mo-yr of birth as random effects evaluated AFC associations with continuous outcomes (P305M, CUML1, CUML1&2, CUMDIM). Multivariable logistic regression assessed AFC associations with removal (sold), mortality, and stillbirth risk in both lactations. Variance inflation factors <5 indicated no significant collinearity. Final models used backward manual stepwise elimination (*P* > 0.05 for main effects and *P* > 0.10 for interactions), with Bayesian information criterion used to select the most parsimonious model while considering fit. Predicted probabilities were derived from logistic regression log-odds for a representative cow reflecting the observed distributions of categorical variables (e.g., PNEU: 0 = 74%, 1 = 17%, 2 = 6%, 3+ = 3%) and mean values of continuous variables unless otherwise stated. Log-odds were exponentiated and then divided by 1 + the exponentiated log-odds to derive the estimated incidence (risk) for logistical regression model outcomes (DeMaris and Selman, 2013). Predicted milk and DIM from mixed models were computed directly from final parameter estimates.

The final dataset included 128,252 cows (239–8,666 per dairy) across 7 AFC cohorts (Table 1). Heifers calving at 22 to 24 mo accounted for 73% of the dataset; the 23-mo cohort was the largest, and the 27-mo cohort the smallest. Herd and mo-yr of birth were significant random effects (*P* < 0.02). Among fixed effects, PNEU, AFC, twinning, and stillbirth at first calving were each significant for all 3 outcomes (*P* < 0.001). The variable DLACT and DLACT × AFC were significant for P305M and CUML1 (*P* < 0.02); for CUML1&2, DLACT was significant (*P* < 0.01) and DLACT × AFC was retained (*P* = 0.07). The categorical variable PNEU showed a dose-response association across all 3 models, with group 0 producing the most milk and group 3+ the least. Singleton versus twin first calving yielded large production differences across all 3 outcomes (*P* < 0.001 for each): +808 kg (P305M), +902 kg (CUML1), and +2,362 kg (CUML1&2). Liveborn versus stillborn effects were similarly large and significant across all outcomes (*P* < 0.001 for each): +962, +1,103, and +2,375 kg, respectively. Although DLACT × AFC was retained, its biological effect was small. Pairwise comparisons using Student's *t*-test showed no differences among DLACT groups 0 to 2 or between groups 3 and 4+ with heifers from younger dams producing modestly more milk (+108, +277, and +481 kg across the 3 outcomes). The AFC optimum differed by outcome (Table 1): P305M peaked at 26 mo, whereas CUML1 and CUML1&2 were both maximized at 23 mo.

**Table 1:** Total counts, milk production, and DIM means (SE) from each of the linear mixed models as predicted for a representative cow within each age at first calving (AFC) category^1^

Item	AFC (mo)						
	21	22	23	24	25	26	27
Number of heifers calving	12,715	35,505	**36,943**	21,137	12,485	6,556	2,911
% of total calving	9.9	27.7	**28.8**	16.5	9.7	5.1	2.3
Projected 305-d milk (kg)	9,108^a^ (235.8)	9,182^a^ (231.9)	9,323^b^ (231.7)	9,310^b^ (232.4)	9,373^b^ (234.1)	**9,392**^b^ (237.4)	9,271^b^ (244.1)
Cumulative milk by 305 d of first lactation (kg)	8,892^a^ (206.5)	9,115^a^ (197.6)	**9,316**^b^ (197.3)	9,242^b^ (198.9)	9,142^bc^ (202.5)	9,044^ac^ (209.8)	8,719^d^ (224.2)
Cumulative milk by 305 d of second lactation (kg)	17,981^ab^ (483.0)	18,133^b^ (462.0)	**18,330**^a^ (461.2)	18,008^ab^ (465.1)	17,561^c^ (473.6)	17,256^c^ (490.7)	15,663^d^ (525.0)
Cumulative DIM to 305 d of second lactation	**514**^a^ (6.8)	510^ab^ (5.9)	507^b^ (5.9)	495^c^ (6.0)	479^d^ (6.4)	468^d^ (7.1)	427^e^ (8.3)

a–e Means within a row with different superscripts differ (*P* < 0.05).

1 Bold text indicates highest numerical (not necessarily significantly different) values at the row level.

For CUMDIM, herd and mo-yr of birth were significant random effects (*P* < 0.01), and PNEU, AFC, twinning, stillbirth (all *P* < 0.001), and DLACT × AFC (*P* = 0.05) were significant. Cumulative DIM was not different across DLACT 0 to 3, but these heifers accumulated 8 to 13 more DIM than those from DLACT 4+ dams (*P* < 0.03). Younger AFC was associated with more cumulative DIM (Table 1).

For first-calving stillbirth, all main effects (herd, AFC, DLACT, mo-yr of birth) were significant (*P* < 0.01) with no interactions. Stillbirth risk increased progressively with DLACT (3.6%–5.4%), whereas risk stratified by AFC showed no consistent directional trend (2.8%–3.9%; Table 2). For second-calving stillbirth, only herd and mo-yr of birth were significant; AFC was retained solely for AFC-specific estimates (Table 2).

**Table 2:** Calculated stillborn, sold, and mortality risks for first lactation and second lactation using transformed parameter estimates from the multivariable logistic regression models as predicted for a representative cow for each age at first calving (AFC) category

Item (%)	AFC (mo)						
	21	22	23	24	25	26	27
First calving stillbirth risk	3.9	3.0	2.9	3.1	3.4	2.9	2.8
Second calving stillbirth risk	2.2	2.1	2.1	2.1	2.4	2.5	2.5
% Sold, first lactation	14.5	15.3	16.1	17.6	20.3	22.7	31.2
% Died, first lactation	2.7	2.8	2.7	3.3	3.5	3.7	4.2
Replacement risk, first lactation	17.2	18.1	18.8	20.9	23.8	26.4	35.4
% Calving to lactation 2	82.8	81.9	81.2	79.1	76.2	73.6	64.6
% Sold, second lactation (through 305 d)	20.8	20.8	21.3	22.4	22.3	21.9	23.3
% Died, second lactation (through 305 d)	3.5	3.4	3.6	3.7	3.9	3.6	5.1
Replacement risk, second lactation (305 d)	24.3	24.2	24.9	26.1	26.2	25.5	28.4

For first-lactation removal risk, all model effects were significant (*P* < 0.001), with no significant interactions detected. Removal risk was markedly elevated for twins (34% vs. 20% for singletons), for calvings following stillbirth (31% vs. 20% for liveborn), and for cows with 3+ PNEU treatments (24% vs. ∼20% for other groups). Variation across DLACT groups was modest (20%–22%), whereas AFC showed the greatest range (17% at 21 mo to 35% at 27 mo; Table 2). Mortality estimates followed a similar pattern, except that mo-yr of birth was not significant. Elevated mortality was observed for twins (4.9% vs. 2.9%), stillbirth (6.5% vs. 2.9%), increasing PNEU severity (2.9%–4.0%), and older AFC (2.7% at 21 mo to 4.2% at 27 mo; Table 2), with modest variation across DLACT groups (2.8%–3.3%). Second-lactation models mirrored first-lactation models, with 2 differences: twinning and stillbirth were defined at second calving, and PNEU was not significant. All remaining effects were significant (*P* < 0.001) with no interactions. Removal risk for twins versus singleton was 36% versus 25%, and stillbirth versus liveborn was 38% versus 25%; DLACT showed no clear directional trend (24%–27%), and AFC ranged less than in first lactation, from 24% (21 mo) to 28% (27 mo; Table 2). For both lactations, percentage sold was calculated by subtracting mortality from total removal risk (Table 2).

The economic model combined analytical results with BW data from a subset of herds to estimate net income across AFC cohorts through 2 lactations. First- and second-lactation sold and died risks (Table 2) served as inputs in a previously described model that follows a cohort through 10 potential lactations, assigning each lactation its own sold and died risks, lactation and dry period length, transition disease risk, and lactation curve, and generates a net present value (**NPV**) as of the first calving for future costs and returns (Overton and Eicker, 2022b). Project-specific production and feed cost results replaced original inputs, and each AFC category used the derived first- and second-lactation removal risks, but original inputs were held constant across AFC levels for third through tenth lactation. The default herd-average replacement rate across all 10 lactations was 34%. Differences in projected productive life, salvage value, and net replacement cost are therefore attributed solely to first- and second-lactation performance. Replacement cost per day equaled NPV average cow cost (heifer cost minus future net salvage value) divided by projected lifetime days (lactating + dry). For example: $1,901 ÷ 1,017 d = $1.87/d.

Key economic inputs reflected current US dairy industry conditions based on consultant interviews and professional experience: milk = $0.44/kg; TMR feed cost = $0.29/kg DM; TMR energy density = 1.72 Mcal of NEL/kg of DM; marginal feed = 0.43 kg of feed/kg of milk; blended calf value = $1,000; replacement cost = $3,500; market cow value = $3.31/kg; interest rate = 8%. A raising cost of $2.75/d was applied from weaning to breeding entry to estimate the cost of delayed AFC, derived from an updated heifer-raising cost calculator (Overton, 2020).

Lactating feed costs had 3 components: (1) maintenance and activity, based on average BW during first lactation (average of first and second calving weights); (2) marginal feed for milk production (0.43 kg of feed/kg of milk); and (3) incremental feed for first-lactation weight gain (5.1 Mcal of NEL/kg of gain; NRC, 2001). Calving weights and first lactation gain by AFC group from 3 farms within the larger dataset that recorded weights at 1 to 3 DIM served as inputs (Table 3). The 24-mo AFC cohort was assigned 615 kg (82% of a 750-kg MBW) and cohort weights varied up or down based on results of the subset of farms. Modeled average BW ranged from 633 kg (21-mo cohort) to 689 kg (27-mo cohort), an increase of approximately 9 kg per AFC mo (Table 3).

**Table 3:** Modeled BW, revenues, costs, and net income predictions through the first 2 potential calvings, a standardized 500-d lactation, and a single 57-d dry period for heifers in age at first calving (AFC) cohorts ranging from 21 to 27 mo^1^

Item	AFC (mo)						
	21	22	23	24	25	26	27
BW at first calving (kg)	581	590	603	615	630	642	656
Incremental weight gain (kg)	105	96	90	84	78	72	66
BW at second calving (kg)	686	686	693	700	708	713	722
Average BW (kg)	633	638	648	657	669	678	689
Total milk value ($)	7,713	7,833	7,974	8,022	8,077	8,133	8,086
Calf value at first calving ($)	961	970	971	969	966	971	972
Calf value at second calving ($)	811	802	795	775	744	717	630
**Total revenue** ($)	**9,484**	**9,605**	**9,740**	**9,765**	**9,788**	**9,822**	**9,688**
Maintenance + activity feed cost ($/d)	2.24	2.25	2.27	2.30	2.33	2.35	2.37
Marginal feed cost (for milk; $/d)	4.31	4.37	4.45	4.48	4.51	4.54	4.51
Feed cost for weight gain ($/d)	0.27	0.24	0.23	0.21	0.20	0.18	0.17
Total feed cost (500-d milking; $)	3,405	3,434	3,477	3,495	3,517	3,535	3,528
Replacement cost ($/d)	1.84	1.85	1.87	1.92	1.98	2.02	2.28
Total replacement cost (557 d; $)	1,023	1,031	1,042	1,071	1,103	1,128	1,270
Additional raising cost ($)	Referent	84	167	251	334	418	502
**Total costs** ($)	**4,428**	**4,548**	**4,686**	**4,817**	**4,954**	**5,081**	**5,300**
**Net income** ($)	**5,057**	**5,057**	**5,054**	**4,948**	**4,833**	**4,741**	**4,388**

1 Bold text indicates total revenue, total costs, and final, net values.

Average milk per cow per day was estimated as CUML1 ÷ CUMDIM. All AFC results were standardized to 500 CUMDIM, a representative midpoint across all cows (Table 1). Total milk value per cohort = average milk/d × 500 d × $0.44/kg. Calf value at first calving = $1,000 × (1 − first-calving stillbirth risk); at second calving = $1,000 × (1 − second-calving stillbirth risk) × proportion calving to lactation 2. Total revenue summed milk value and both calf values across the 500-d lactation and 57-d dry period (full dataset average).

Total feed cost per cohort summed the 3 daily feed cost components × 500 d. Second-lactation weight gain was excluded, as BW differences across AFC cohorts narrow substantially by that point. Dry cow costs were assumed equal across cohorts and omitted. Total replacement cost = replacement cost/d × 557 d (500 lactating + 57 dry). Additional raising cost = $2.75 × extra days from delayed AFC. Total cost = feed + replacement + additional raising costs. Net income per AFC group = total revenue − total cost (Table 3).

Consistent with other large datasets, P305M and estimated daily milk were highest at older AFC (Eastham et al., 2018; Van Eetvelde et al., 2020). However, the confounding influence of body size and maturity must be considered as older heifers are generally larger, have greater feed intake capacity, and face less physiological demand for growth. Delayed calving is not the solution; rather, management should target modestly improved growth rates, particularly during the efficient early-life and prepubertal periods. Well-grown heifers achieving lean tissue gain without excess fat deposition can calve young while sustaining high first-lactation production (Van Amburgh et al., 1998; Macdonald et al., 2005; Kusaka et al., 2023).

A notable finding was the apparent divergence in “optimal” AFC by production metric. Consultants often advocate moderately delayed AFC (24–25 vs. 22–23 mo), which appears justified when evaluating P305M. However, P305M is subject to culling bias: although heifers calving at 25 to 26 mo had higher projected production, fewer survived to the end of first lactation. When CUML1 was the outcome, peak production shifted to 23 mo, driven by greater survival, and was reinforced by CUML1&2 results. Cows leaving the herd prematurely contribute no additional milk; the cumulative approach captures this reality. Thus, basing AFC decisions on P305M alone overlooks the survival disadvantage associated with older calving ages.

Cumulative production results suggest minimal economic differences among heifers calving at 21 to 23 mo, but a pronounced decline in net income emerged beginning at 24 mo, with losses increasing through 27 mo. Although older heifers generated more milk revenue, this was offset by lower calf revenue, higher maintenance feed costs, higher replacements costs, and greater raising costs.

Maintenance feed costs increased by $0.023/d per mo of AFC delay, driven by BW differences during first lactation. Replacement cost per day ranged from $1.84/d (21 mo) to $2.28/d (27 mo), driven primarily by higher first-lactation replacement rates (17% to 35% across cohorts). Second-lactation replacement risk followed the same directional pattern but with smaller differences. Combined, these higher replacement risks reduced projected lifetime days from 1,039 d (21 mo) to 932 d (27 mo), assuming no differences in replacement risk beyond second lactation. Additional raising costs of $2.75/d ($84/mo) substantially eroded, and ultimately eliminated, the revenue advantages of the 24- to 26-mo AFC cohort.

Younger-calving heifers generally achieve comparable or better reproductive performance than older counterparts. Cooke et al. (2013) found conception rate to first service was highest in the youngest AFC group and lowest in cows calving after 30 mo, whereas Kusaka et al. (2023) found no significant reproductive differences among AFC groups in cows surviving to third calving, suggesting adequate calving weight may be the critical factor rather than AFC itself. Consistent with these findings, ancillary analyses from the current dataset revealed small but significant improvements in the proportion pregnant by 250 DIM for younger AFC cohorts, contributing to the lower replacement risks observed in these groups.

A younger AFC also shortens the generation interval, accelerates genetic progress, increases calves produced per unit of time, and enables earlier positive cash flow—advantages further amplified by expanded genomic selection. Although not directly quantified here, each has meaningful potential to strengthen the economic case for earlier AFC. Genomic data were excluded due to inconsistent availability across herds, but some apparent AFC effects observed may reflect underlying genetic influences. Lauber and Fricke (2023) grouped primiparous cows by BW at first calving and found that heavier heifers had greater genomic merit for milk but lower genomic merit for daughter pregnancy rate—a pattern consistent with well-established correlations between body size, milk production, and fertility. Future research should use a prospective design with complete genomic data and BW recorded at each calving to better disentangle the effects of size, genetic potential, and AFC.

Several limitations should be noted. This study examined associations, not causation, and BW were available from only a subset of farms. Notably, Costigan et al. (2025) found that BW at first calving, rather than AFC itself, had a more durable influence on milk production across the first 3 lactations, suggesting that size and genetic potential at calving may be stronger determinants of performance than age alone, particularly in confinement systems. Although these results should not be used to set definitive AFC targets, they meaningfully support the economic case for younger AFC, given the additional raising and replacement costs associated with delayed first calving.
